# Petal-like g-C_3_N_4_-enabled electrochemical immunosensor for urinary cytokeratin 19 assessment in endometriosis: a proof-of-concept pilot study

**DOI:** 10.3389/fbioe.2026.1905091

**Published:** 2026-08-27

**Authors:** Yangyang Wang, Bin Liu, Xiaohua Yu, Liu Liu, Xiaoying Jin, Yan Rong, Songying Zhang

**Affiliations:** 1 Assisted Reproduction Unit, Department of Obstetrics and Gynecology, Sir Run Run Shaw Hospital, Zhejiang University School of Medicine, Hangzhou, China; 2 Zhejiang Key Laboratory of Precise Protection and Promotion of Fertility, Hangzhou, China; 3 Zhejiang Provincial Clinical Research Center for Reproductive Health and Disease, Hangzhou, China; 4 School of Biomedical Engineering, Shanghai Jiao Tong University, Shanghai, China; 5 Shanghai 860 Technology Development Company, Shanghai, China; 6 Key Laboratory of Motor System Disease Research and Precision Therapy of Zhejiang, Orthopedics Research Institute of Zhejiang University, Second Affiliated Hospital Zhejiang University School of Medicine, Hangzhou, Zhejiang Province, China

**Keywords:** endometriosis, noninvasive biomarker, petal-like g-C_3_N_4_, pilot clinical evaluation, portable electrochemical immunosensor, urinary cytokeratin 19

## Abstract

Endometriosis is a debilitating, estrogen-dependent gynecological disorder characterized by profound diagnostic delays due to clinical reliance on invasive laparoscopy. Developing noninvasive, point-of-care biomarker assessment remains a critical yet unmet medical need. Here, we report an integrated electrochemical immunosensing platform (“Flower-CN-EC”) based on a structurally engineered, petal-like graphitic carbon nitride (g-C_3_N_4_) framework for the sensitive detection of urinary cytokeratin 19 (CK-19)-a promising candidate biomarker reflecting endometriosis-associated epithelial activity. The 3D hierarchical architecture of curled nanosheets provides an optimized microenvironment that facilitates unhindered mass transport and minimizes steric hindrance during covalent antibody immobilization. To bridge laboratory sensing with decentralized clinical translation, the sensor was coupled with a customized visual signal-processing interface featuring automated polynomial baseline correction. The sensor showed an excellent log-linear response to CK-19 (10 pg/mL-100 ng/mL), a low detection limit of 3.24 pg/mL, outstanding batch reproducibility (relative standard deviation 2.31%, n = 8), and exceptional ambient storage stability over 5 weeks. In a pilot clinical evaluation (n = 21), the platform successfully differentiated patients with histologically confirmed endometriosis from healthy controls with high statistical significance (*p* = 0.0011) and AUC of 0.899. These findings establish a robust, noninvasive proof-of-concept paradigm, paving the way for low-cost, decentralized endometriosis screening and longitudinal treatment monitoring.

## 1 Introduction

**SCHEME 1:**
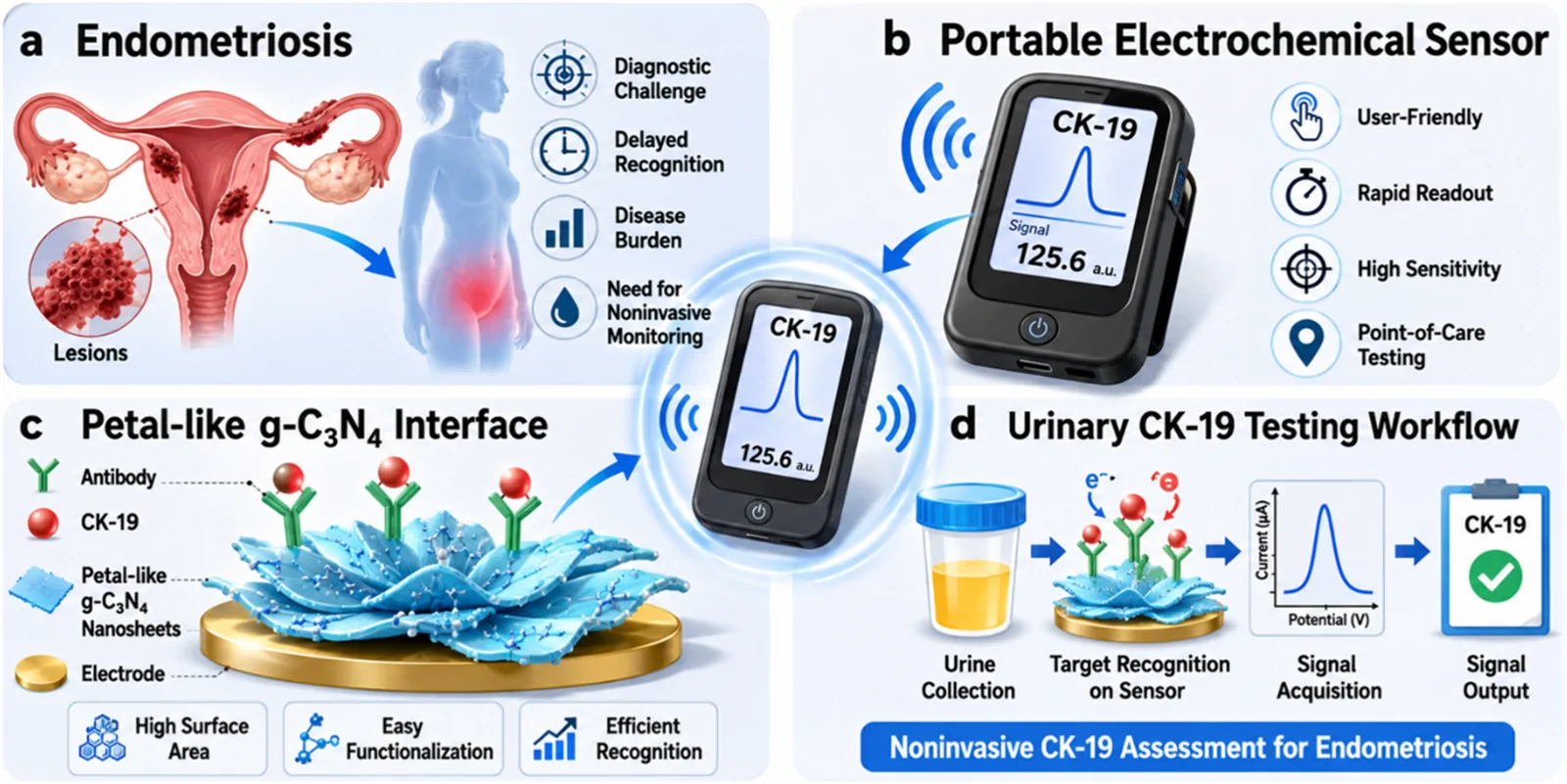
Schematic overview of the petal-like g-C_3_N_4_-enabled urinary CK-19 sensing strategy for endometriosis-related biomarker assessment. **(a)** Endometriosis is associated with substantial clinical and disease-management challenges, including diagnostic difficulty, delayed clinical recognition, disease burden, and the need for noninvasive monitoring approaches. **(b)** The portable electrochemical sensor format is intended to support user-friendly operation, rapid signal readout, high analytical sensitivity, and future point-of-care-oriented biomarker testing. **(c)** The petal-like g-C_3_N_4_ sensing interface provides an accessible nanosheet-based architecture with a high specific surface area and facile functionalization potential, thereby supporting antibody immobilization and CK-19 recognition at the electrode interface. **(d)** The proposed urinary CK-19 testing workflow includes urine collection, target recognition on the modified electrochemical sensor, signal acquisition, and signal output for noninvasive endometriosis-related biomarker assessment. This scheme illustrates the conceptual framework of the platform and its potential for future portable testing applications.

Endometriosis, EMS, is an estrogen-dependent chronic inflammatory gynecological disorder characterized by the presence of endometrial-like glands and stroma outside the uterine cavity (Scheme 1a) (Tokushige et al., 2011; Lamceva et al., 2023; Vissers et al., 2024). It affects approximately 10%–15% of women of reproductive age and is more prevalent among women presenting with infertility (Rahmioglu et al., 2023; Barnard et al., 2024). Common clinical manifestations include progressive dysmenorrhea, chronic pelvic pain, dyspareunia, and infertility, which can substantially impair quality of life and reproductive health. However, clinical assessment remains challenging because these symptoms often overlap with those of pelvic inflammatory disease, irritable bowel syndrome, adenomyosis, and other benign gynecological or gastrointestinal conditions. Although imaging and clinical evaluation play important roles in disease assessment, definitive confirmation still commonly relies on laparoscopic visualization and histopathological examination, which are invasive, costly, and unsuitable for repeated follow-up or home-based monitoring (Lamceva et al., 2023; Barnard et al., 2024; Vissers et al., 2024). Laparoscopy with histopathological confirmation remains the diagnostic reference standard; however, its invasive nature, high cost, and dependence on surgical expertise preclude its use for repeated monitoring, population-based screening, or early-stage detection. Noninvasive imaging modalities, including transvaginal ultrasound and magnetic resonance imaging, offer valuable adjunctive information, but their diagnostic performance varies considerably according to disease location, lesion type, and operator experience, with notable limitations for detecting superficial peritoneal lesions and deep infiltrating nodules in certain anatomical sites. Consequently, imaging alone cannot reliably exclude endometriosis (Lamceva et al., 2023; Barnard et al., 2024; Vissers et al., 2024). Serum biomarkers, most notably CA125, have been extensively investigated, yet they exhibit insufficient sensitivity and specificity for standalone clinical use, particularly in early-stage or atypical presentations. While multi-marker panels and emerging circulating molecules have shown promising trends, they remain largely investigational and lack standardized validation. Urinary biomarkers offer the distinct advantage of completely noninvasive collection; however, existing immunoassays for urinary cytokeratin-19 fragments have demonstrated limited clinical sensitivity, highlighting the critical need for highly sensitive and robust detection platforms capable of reliably measuring low-abundance targets in complex urine matrices. Therefore, sensitive, convenient, and noninvasive approaches are needed to support auxiliary assessment, treatment monitoring, and recurrence surveillance in endometriosis.

Electrochemical biosensors are attractive platforms for noninvasive biomarker analysis because they offer rapid signal readout, compact instrumentation, low cost, and compatibility with point-of-care testing, point-of-care testing (POCT), formats (Xu et al., 2020; Liu et al., 2021; Wang et al., 2022b). Compared with serum, urine is particularly suitable for patient-friendly monitoring because it can be collected noninvasively without venipuncture or specialized clinical personnel. Cytokeratin 19, CK-19, is an epithelial-derived intermediate filament protein that has been investigated as a protein biomarker in several diseases (Kalomenidis et al., 2004; Hillas et al., 2008; Yang et al., 2019). Previous studies have reported CK-19-related urinary signals in women with endometriosis (Carson and Kallen, 2021), suggesting that urinary CK-19 or CK-19-derived immunoreactive fragments may provide a noninvasive window into endometriosis-associated epithelial activity. Nevertheless, CK-19 should currently be regarded as a candidate urinary biomarker rather than a stand-alone diagnostic marker for endometriosis. Because urinary protein levels are often low and urine contains complex matrix components that may interfere with biomolecular recognition and electrochemical readout, a sensing interface with high sensitivity, stable antibody immobilization, and anti-interference capability is required.

To date, only a limited number of electrochemical biosensors have been explored for endometriosis-associated biomarker detection, predominantly targeting serum CA125 or a few other proteins using conventional electrode materials such as gold nanoparticles or carbon nanotubes. However, most of these reported approaches still rely on rigid electrode substrates, relatively complex fabrication procedures, or bulky instrumentation, which collectively restrict their translation toward patient-friendly, point-of-care applications. Moreover, no electrochemical immunosensor has yet been reported for the detection of urinary CK-19—a promising noninvasive biomarker candidate reflecting endometriosis-associated epithelial activity—using a structurally engineered 3D hierarchical sensing interface. Carbon nitride, g-C_3_N_4_, is a metal-free polymeric semiconductor with a layered structure, high chemical stability, good biocompatibility, and abundant nitrogen-containing groups (Chen et al., 2019; Yu et al., 2020; Pasupuleti et al., 2022). These physicochemical properties make g-C_3_N_4_ suitable for electrode modification and biomolecule immobilization. Compared with bulk g-C_3_N_4_, petal-like g-C_3_N_4_ obtained through solvothermal-assisted thermal exfoliation can form curled and folded nanosheet assemblies, which may enlarge the accessible surface area, facilitate mass transfer, and expose additional sites for antibody attachment (Pasupuleti et al., 2022; Pasupuleti et al., 2023). Therefore, petal-like g-C_3_N_4_ represents a promising interface material for constructing electrochemical immunosensors with enhanced analytical performance. g-C_3_N_4_-based electrochemical sensors have been extensively explored for detecting diverse disease biomarkers (Chen et al., 2019; Yu et al., 2020; Pasupuleti et al., 2022), including neuron-specific enolase, alpha-fetoprotein, prostate-specific antigen, carcinoembryonic antigen, microRNAs, and viral proteins. However, most reported sensors employ conventional bulk or sheet-like g-C_3_N_4_ morphologies and focus predominantly on serum-based cancer markers. In this context, recent nanohybrid-based electrochemical sensors, such as the multicomponent 2D/0D MoS_2_/g-C_3_N_4_/carbon-dot platform reported by Parambil et al. for label-free hCG detection, have demonstrated the value of rational nanointerface engineering for improving charge transfer, antibody loading, and low-level biomarker detection (Ho et al., 2021; Parambil et al., 2025). Different from these prior nanohybrid-based strategies, the present Flower-CN-EC platform employs a 3D petal-like g-C_3_N_4_ architecture to enhance antibody immobilization and target recognition, integrates this interface with a flexible and reproducible screen-printed electrode and portable signal-processing system, and provides the first proof-of-concept clinical evaluation of urinary CK-19 detection for endometriosis, thereby addressing a distinct unmet need in noninvasive gynecological biomarker assessment.

In this study, we developed a portable petal-like g-C_3_N_4_-based electrochemical immunosensor, termed Flower-CN-EC, for urinary CK-19 assessment in endometriosis, Schemes 1b–d. The sensor integrates a flexible screen-printed three-electrode substrate, a petal-like g-C_3_N_4_-modified sensing interface, anti-CK-19 antibody immobilization, and a portable signal readout and visualization system. We systematically evaluated the material characteristics, stepwise electrode modification, analytical performance, selectivity, reproducibility, stability, and preliminary performance in human urine samples. This proof-of-concept study provides initial evidence that petal-like g-C_3_N_4_-enabled electrochemical sensing may support noninvasive urinary CK-19 assessment for endometriosis-related biomarker analysis, while also highlighting the need for larger, standardized, and independently validated clinical studies before diagnostic utility can be established.

## 2 Materials and methods

### 2.1 Chemicals and materials

Urea, melamine, ethylene glycol, and absolute ethanol were purchased from Sinopharm Chemical Reagent Co., Ltd. (Shanghai, China). Cytokeratin 19 (CK-19) monoclonal capture antibody, recombinant CK-19 protein, bovine serum albumin (BSA), 1-ethyl-3-(3-dimethylaminopropyl) carbodiimide (EDC), N-hydroxysuccinimide (NHS), and phosphate-buffered saline (PBS, 0.1 M, pH 7.4 unless otherwise stated) were obtained from Sigma-Aldrich (United States). Carcinoembryonic antigen (CEA), carbohydrate antigen 125 (CA125), and CD44 protein were purchased from Abcam (United Kingdom). Glucose was obtained from Aladdin Industrial Corporation (Shanghai, China). All reagents were of analytical grade and used without further purification. Deionized water (18.2 MΩ cm) was used throughout the experiments. Human urine samples were collected under the approved institutional ethics protocol after written informed consent was obtained.

### 2.2 Synthesis of petal-like g-C_3_N_4_

Petal-like g-C_3_N_4_ was synthesized using a solvothermal-assisted thermal exfoliation method (Chen et al., 2019; Yu et al., 2020; Pasupuleti et al., 2022). Briefly, 2 g of melamine was dispersed in 40 mL of ethylene glycol under vigorous stirring for 30 min. The mixture was transferred into a 100 mL Teflon-lined stainless-steel autoclave and heated at 180 °C for 12 h under static conditions without stirring. After the reaction, the autoclave was allowed to cool naturally to room temperature. The resulting white precipitate was collected by centrifugation at 8,000 rpm for 10 min at 4 °C, washed three times with deionized water and absolute ethanol, and then dried at 60 °C for 12 h in a vacuum oven. The obtained precursor was then calcined in a muffle furnace at 550 °C for 2 h with a ramp rate of 5 °C/min under air atmosphere. The final petal-like g-C_3_N_4_ powder was collected and stored in a desiccator before use. Nitrogen adsorption-desorption isotherms were measured at 77 K using a surface area analyzer (ASAP 2460, Micromeritics).

### 2.3 Fabrication of Flower-CN-EC portable sensors

Flexible electrochemical sensors (Flower-CN-EC) were fabricated on polyethylene terephthalate (PET) substrates using screen-printing (Roychoudhury et al., 2016; Mariani et al., 2018; Zhang et al., 2020). Each sensor contained a three-electrode configuration consisting of a carbon working electrode, a carbon counter electrode, and a printed reference electrode. The working electrode was a circular disk with a diameter of 3 mm. After printing, the electrodes were dried at 80 °C for 30 min. Subsequently, 5 µL of petal-like g-C_3_N_4_ dispersion (1 mg/mL in deionized water, freshly sonicated for 30 min to ensure uniform suspension) was drop-cast onto each working electrode using a calibrated micropipette and dried under ambient conditions (25 °C ± 2 °C, 40%–50% relative humidity) for 2 h. An arrayed fabrication layout was used to produce 8–12 sensors per batch. For quality control, each batch underwent visual inspection for coating uniformity and electrochemical verification by cyclic voltammetry (n = 3 randomly selected sensors per batch) to confirm consistent baseline responses; only batches with a relative standard deviation below 5% in peak current were accepted for downstream antibody modification. Fabricated sensors were stored in a clean, dust-free container at room temperature before biochemical modification.

### 2.4 Electrode modification and antibody immobilization

Before antibody immobilization, g-C_3_N_4_-modified working electrodes were treated with oxygen plasma (30 W, 1 min) to increase the density of reactive oxygen-containing groups on the modified interface. For antibody immobilization, 10 µL of freshly prepared solution containing 10 μg/mL anti-CK-19 capture antibody, 20 mM EDC, and 10 mM NHS in PBS (0.1 M, pH 6.0) was added to each working electrode and incubated for 2 h at 37 °C in a humidified chamber. This EDC/NHS-assisted step was used to enhance antibody immobilization on the plasma-treated g-C_3_N_4_ interface, likely through covalent coupling involving oxygen-containing groups introduced during plasma activation, together with nonspecific adsorption and interfacial interactions. Electrodes were gently rinsed with PBS to remove unbound antibody. Then, 10 µL of 1% (w/v) BSA was applied for 30 min at 37 °C to block residual nonspecific adsorption sites. After each step, electrodes were thoroughly washed with PBS. The prepared biosensors were stored at 4 °C for routine use, whereas the storage-stability test was performed under the specified ambient conditions.

### 2.5 Electrochemical measurements and sensor characterization

The flexible Flower-CN-EC sensor was analyzed for current signal recording using a miniaturized portable circuit board. The flexible sensor architecture and visual signal-processing interface were developed as a prototype framework for future integration into portable or point-of-care formats. Cyclic voltammetry (CV) was conducted in 5 mM [Fe(CN)_6_]^3-^/^4-^ containing 0.1 M KCl over the potential range from −0.25 V to +0.65 V at a scan rate of 60 mV/s. Electrochemical impedance spectroscopy (EIS) was recorded in the same redox-probe solution over a frequency range of 0.1 Hz–100 kHz with an AC amplitude of 5 mV at open-circuit potential. For quantitative CK-19 detection, recombinant CK-19 solutions (10 pg/mL to 100 ng/mL in PBS or diluted urine) were incubated on the modified electrode for 30 min at 37 °C. After rinsing with PBS, differential pulse voltammetry (DPV) was performed from −0.20 V to +0.65 V with a pulse amplitude of 50 mV and a scan rate of 20 mV/s in 5 mM [Fe(CN)_6_]^3-^/^4-^ containing 0.1 M KCl. Peak current was used for signal analysis. Long-term signal stability was evaluated by storing sensors under ambient conditions and measuring their response weekly for five weeks. Selectivity was assessed by comparing the DPV response to CK-19 (10 ng/mL) with those to selected potentially interfering species, including CEA, CA125, CD44, BSA, glucose, uric acid, and CYFRA 21-1. Mixed solutions containing CK-19 and representative interferents were also tested to evaluate the anti-interference capability of the sensor under coexisting-analyte conditions.

### 2.6 Clinical sample collection and analysis

This study was approved by the institutional ethics committee, and all participants provided written informed consent. A total of 21 urine samples were collected, including 10 from healthy female volunteers and 11 from patients with endometriosis confirmed by laparoscopy and histopathology. Midstream urine samples were collected in sterile containers, centrifuged at 3,000 rpm for 10 min at 4 °C to remove cellular debris, and stored at −80 °C until analysis (Tokushige et al., 2011; Ray and Steckl, 2019; Nunes et al., 2021). Before measurement, samples were thawed and diluted 1:1 with PBS (0.1 M, pH 7.4). Diluted urine was incubated on the Flower-CN-EC sensor for 30 min at 37 °C, and DPV signals were recorded as described above. Each sample was measured in two independent replicates, and the average peak current was used for statistical analysis. For comparative validation, urinary CK-19 levels in the same clinical samples were determined using a commercial ELISA kit following the manufacturer’s instructions, and the measured concentrations were correlated with the Flower-CN-EC sensor-derived signals. Given the pilot nature of the cohort, the clinical analysis was designed to explore feasibility rather than to establish a diagnostic cutoff. This sample size (n = 21) is consistent with published proof-of-concept electrochemical biosensor and endometriosis urinary biomarker pilot studies, which typically employ small cohorts for initial feasibility assessment.

### 2.7 Statistical analysis

Statistical analysis was performed using SPSS 26.0 or GraphPad Prism 9.0. Sensor reproducibility and stability are expressed as relative standard deviation (RSD). Normality of clinical-sample data was assessed using the Shapiro-Wilk test. Between-group comparisons were performed using an unpaired Student’s t-test for normally distributed data or the Mann-Whitney U test for non-normally distributed data. Receiver operating characteristic (ROC) curve analysis was performed to estimate the discriminatory performance of the sensor signal, and the area under the curve (AUC) was reported where applicable. Owing to the pilot sample size and the absence of an independent validation cohort, cutoff estimation was considered exploratory. A two-sided p value <0.05 was considered statistically significant.

## 3 Results

### 3.1 Material characterization

**FIGURE 1:**
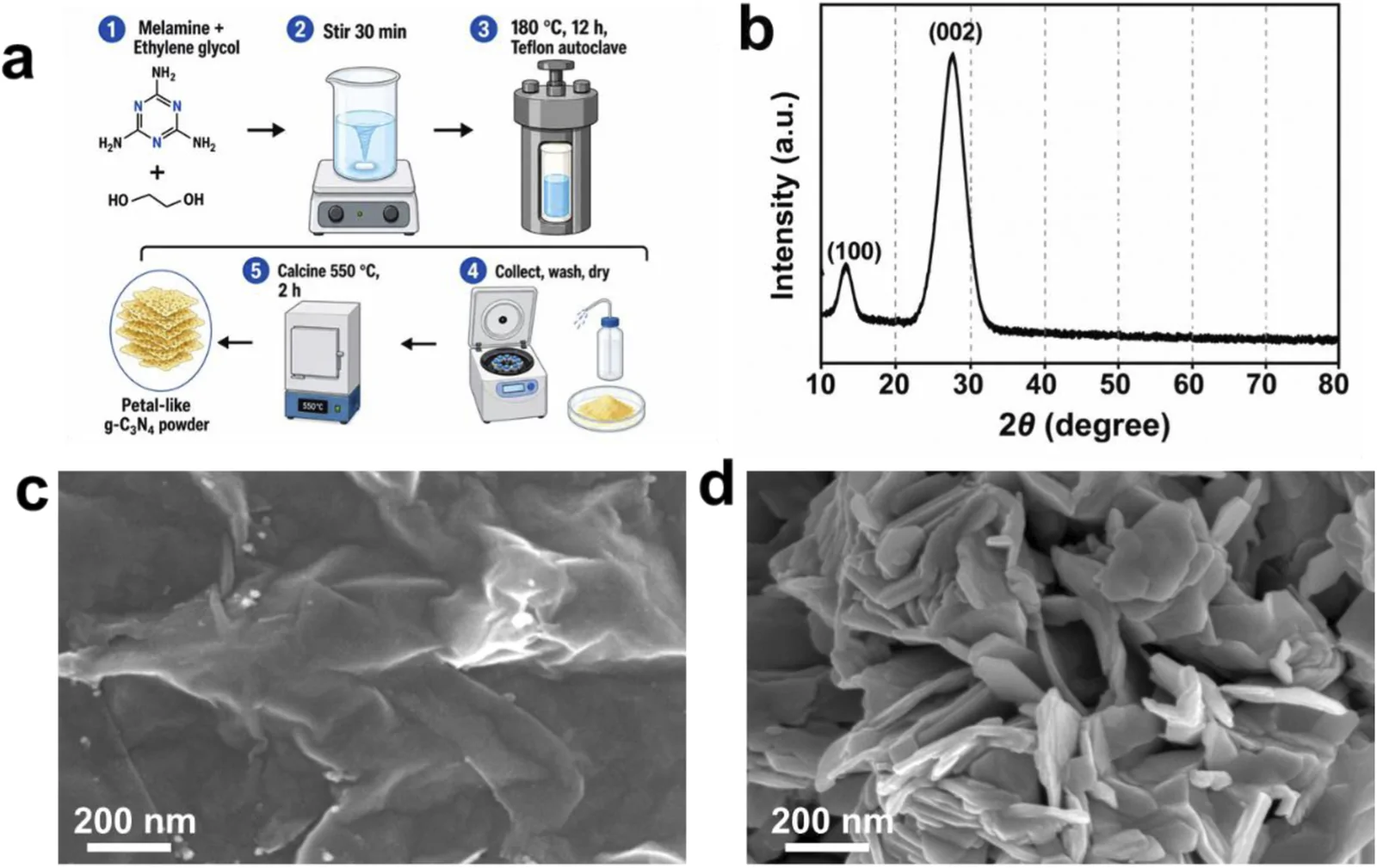
Preparation and characterization of petal-like g-C_3_N_4_. **(a)** Schematic illustration of the solvothermal-assisted thermal treatment process used to prepare petal-like g-C_3_N_4_. Melamine was dispersed in ethylene glycol, stirred for 30 min, treated in a Teflon-lined autoclave at 180 °C for 12 h, collected, washed, dried, and subsequently calcined at 550 °C for 2 h to obtain petal-like g-C_3_N_4_ powder. **(b)** X-ray diffraction, XRD, pattern of the prepared material showing characteristic diffraction peaks assigned to the (100) and (002) planes of graphitic carbon nitride, which are associated with in-plane structural packing and interlayer stacking, respectively. **(c, d)** Scanning electron microscopy, SEM, images showing the morphological evolution from partially assembled sheet-like structures to a hierarchical petal-like architecture composed of curled, folded, and stacked nanosheets. This nanosheet-based morphology provides an accessible structural interface for subsequent electrode modification and antibody immobilization.

Petal-like g-C_3_N_4_ was prepared using a scalable solvothermal-assisted thermal exfoliation strategy, as illustrated in Figure 1a. X-ray diffraction (XRD) analysis confirmed the characteristic structural features of graphitic carbon nitride, with diffraction peaks corresponding to the (100) and (002) crystal planes (Figure 1b). These peaks are associated with the in-plane structural packing and interlayer stacking of g-C_3_N_4_, respectively, indicating successful formation of the target material.

Scanning electron microscopy (SEM) was used to examine the morphological evolution during material preparation. The intermediate product exhibited partially assembled sheet-like structures (Figure 1c), whereas the final product showed a distinct hierarchical petal-like morphology composed of curled and folded nanosheets (Figure 1d). Meanwhile, Transmission Electron Microscope (TEM) analysis of the C_3_N_4_ lattice (Supplementary Figure S1, 0, 0.322 nm) revealed consistency with reported literature (Fu et al., 2018), effectively demonstrating the successful synthesis of the layered material. Furthermore, N_2_ adsorption-desorption measurements (Supplementary Figure S2) showed that the petal-like g-C_3_N_4_ exhibited a higher BET specific surface area than bulk g-C_3_N_4_, with values of 72.3 and 51.2 m^2^/g, respectively. This approximately 41% increase in specific surface area indicates that the curled nanosheet-based architecture provides more accessible interfacial sites for subsequent electrode modification and antibody immobilization. In addition, comparative DPV analysis (Supplementary Figure S3) showed a stronger redox response at the petal-like g-C_3_N_4_-modified electrode than at the bulk g-C_3_N_4_-modified electrode, supporting more favorable interfacial electron-transfer behavior. Together, these results indicate that the open 3D hierarchical morphology can enlarge the electrochemically accessible surface area and facilitate redox-probe diffusion through less restricted mass-transport pathways. Crucially, the highly crumpled surface minimizes steric hindrance during the subsequent immobilization of anti-CK-19 antibodies and facilitates efficient target capture, thereby establishing a structurally optimized interface for high-performance biosensor construction. Notably, this petal-like architecture differs substantially from conventional bulk or sheet-like g-C_3_N_4_ morphologies reported in most electrochemical sensors, offering a structurally optimized interface with enhanced surface area and mass transport for biosensing applications.

### 3.2 Fabrication and batch reproducibility of Flower-CN-EC sensors

**FIGURE 2:**
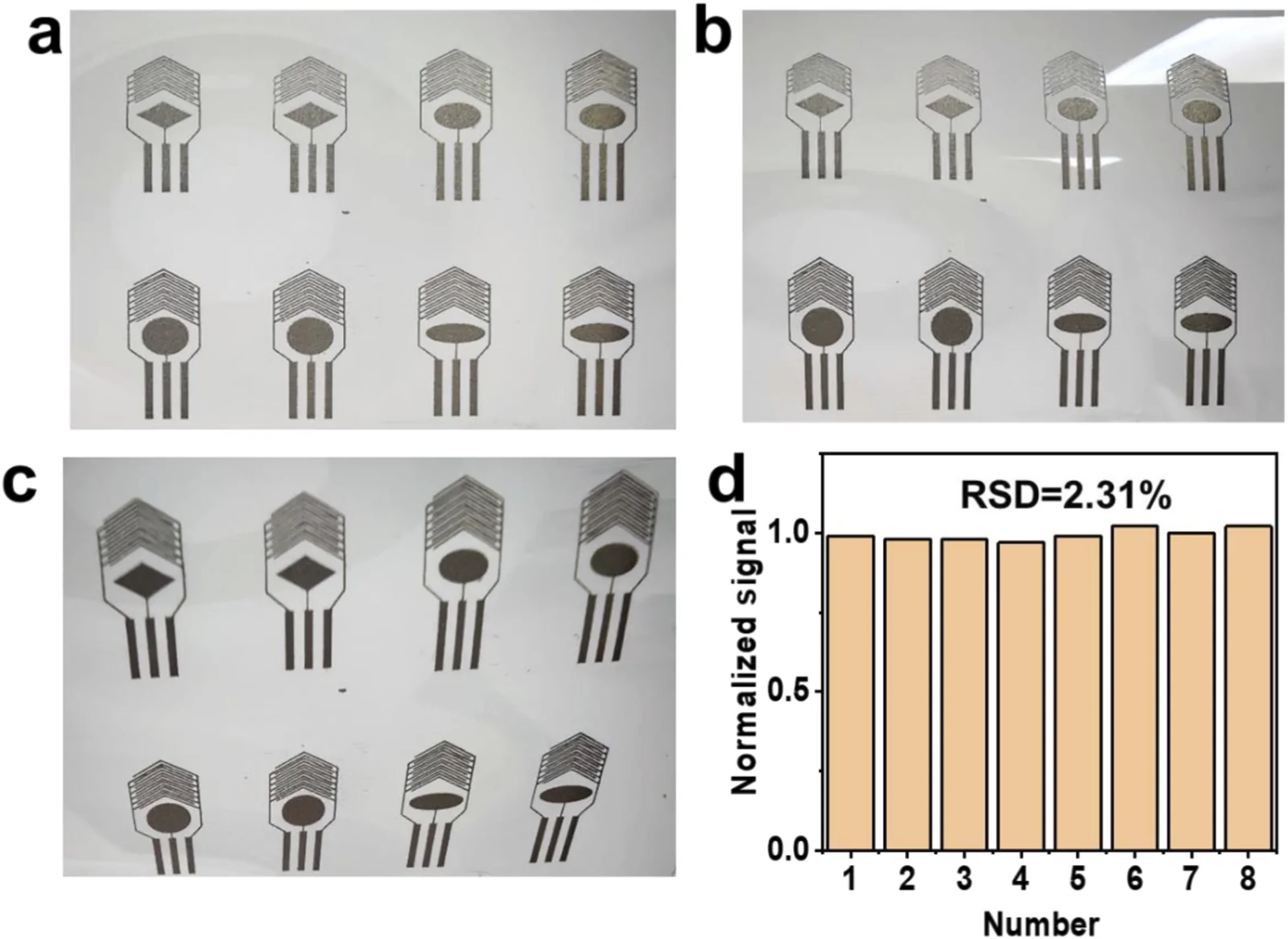
Fabrication format, flexibility, and inter-sensor reproducibility of Flower-CN-EC sensors. **(a, b)** Photographs of arrayed Flower-CN-EC sensors fabricated on flexible PET substrates, showing the batch-prepared screen-printed three-electrode format. **(c)** Photograph of the sensors under a bent configuration, illustrating the mechanical flexibility of the PET-based sensor format. **(d)** Electrochemical response reproducibility of eight independently prepared sensors measured under identical conditions. The relative standard deviation, RSD, was 2.31%, indicating good inter-sensor reproducibility of the fabrication process under the tested conditions.

To transition from material synthesis to scalable device fabrication, petal-like g-C_3_N_4_ was used as the sensing-interface material to fabricate flexible Flower-CN-EC sensors on PET substrates (Figure 2a). The resulting devices were compact, lightweight, and compatible with portable electrochemical instrumentation (Figures 2b,c). The arrayed sensor format enabled multiple devices to be prepared in parallel, supporting batch fabrication and potential practical deployment.

Batch reproducibility was evaluated using eight independently prepared sensors under identical testing conditions. The sensors exhibited consistent electrochemical responses, with a relative standard deviation (RSD) of 2.31% (Figure 2d)—a value that compares favorably with reported RSDs in the electrochemical immunosensor literature (typically ranging from 2.8% to 8%) (Mehta et al., 2024), placing our platform at the superior end of current state-of-the-art performance. This minimal inter-sensor variation demonstrates the high fidelity and robustness of the fabrication protocol, confirming that the structural integrity of the Flower-CN-EC interface is preserved during batch manufacturing–a critical prerequisite for decentralized POCT. Additionally, we performed spike recovery analysis on Flower-CN-EC and found that the RSD of the recovery efficiencies for five replicates was 3.48% (Supplementary Table S1), indicating that Flower-CN-EC exhibits favorable recovery performance and stability.

### 3.3 Functionalization for Flower-CN-EC

**FIGURE 3:**
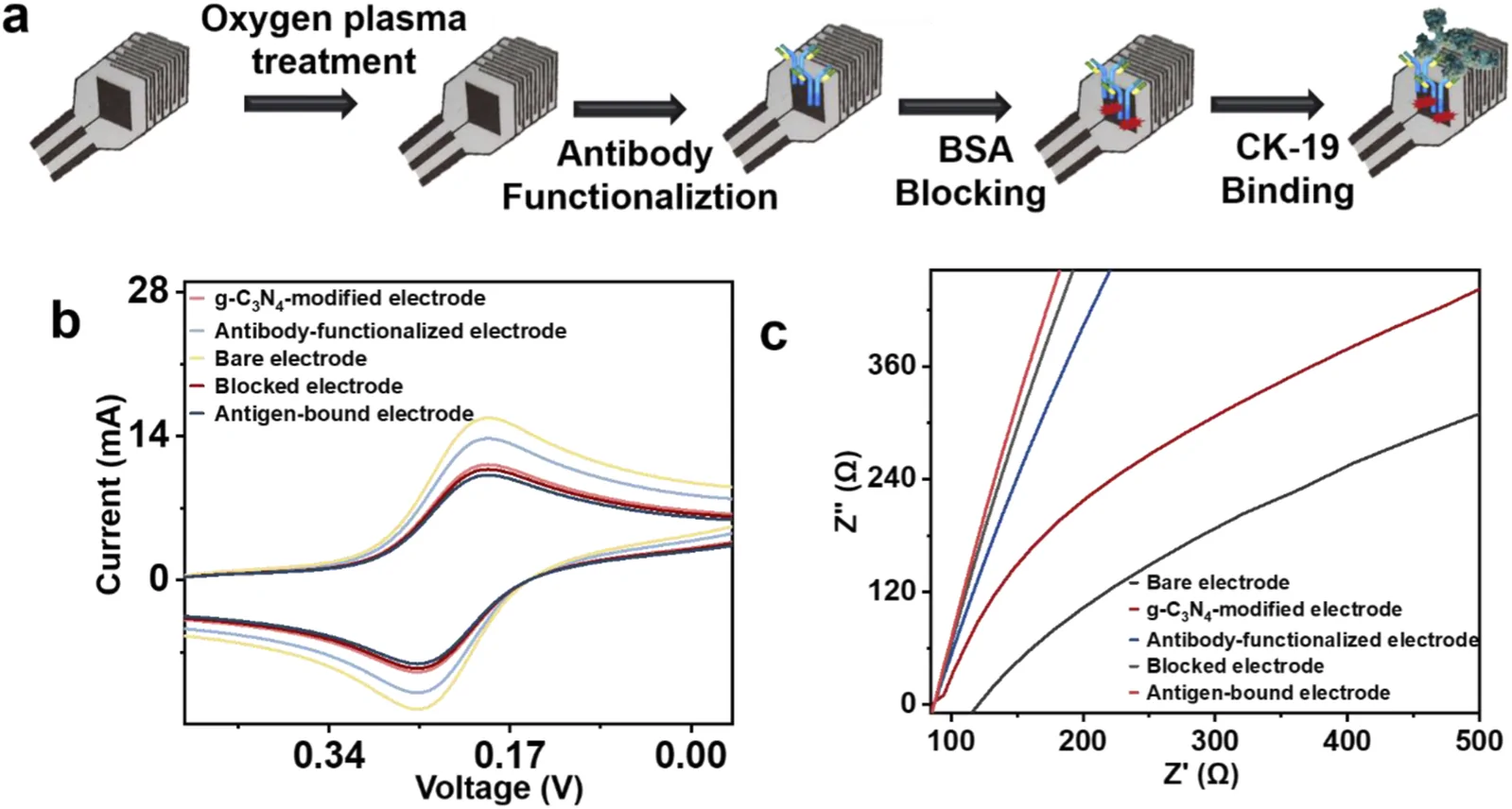
Stepwise fabrication and electrochemical characterization of the Flower-CN-EC biosensing interface. **(a)** Schematic illustration of the sequential modification procedure on the working electrode, comprising petal-like g-C_3_N_4_ deposition, oxygen-plasma treatment, EDC/NHS-assisted anti-CK-19 antibody immobilization, BSA blocking, and final CK-19 antigen capture. **(b)** Cyclic voltammetry (CV) and **(c)** electrochemical impedance spectroscopy (EIS) responses recorded at five consecutive interfacial states: bare electrode, g-C_3_N_4_-modified electrode, antibody-functionalized electrode, BSA-blocked electrode, and antigen-bound electrode. The progressive decrease in redox peak current and the corresponding stepwise increase in charge-transfer resistance (Rct) support the successful layer-by-layer construction of the biorecognition interface and the gradually inhibited interfacial electron transfer during electrode fabrication.

To ensure the specificity of Flower-CN-EC for the detection of CK-19, targeted functionalization modifications are essential. As schematically depicted in Figure 3a, the working electrode was subjected to five sequential modification steps: petal-like g-C_3_N_4_ deposition, oxygen-plasma treatment, EDC/NHS-assisted anti-CK-19 antibody immobilization, BSA blocking, and final CK-19 antigen capture. Cyclic voltammetry (CV) and electrochemical impedance spectroscopy (EIS) were employed to validate each step of this fabrication process.

In the CV measurements (Figure 3b), the redox peak current of the [Fe(CN)_6_]^3-^/^4-^ probe decreased progressively across the five consecutive interfacial states: bare electrode, g-C_3_N_4_-modified electrode, antibody-functionalized electrode, BSA-blocked electrode, and antigen-bound electrode. This sequential attenuation is consistent with the gradual formation of the g-C_3_N_4_ layer and subsequent insulating biomolecular layers, which increasingly hinder interfacial electron transfer. After CK-19 incubation, a further decrease in current was observed, supporting the formation of the anti-CK-19/CK-19 immunocomplex on the electrode surface. Consistently, EIS analysis (Figure 3c) showed a stepwise increase in charge-transfer resistance (Rct) after each modification step. Representative SEM images of the antibody-functionalized and BSA-blocked electrodes further confirmed that the modified sensing layer was retained on the electrode surface. Because the antibody, BSA, and antigen layers are ultrathin and conformal biomolecular films, their stepwise differences are difficult to resolve clearly by morphology-based characterization alone. In contrast, the progressive decrease in CV peak current and the corresponding increase in EIS Rct provide sensitive functional evidence for the successful layer-by-layer assembly of the biosensing interface, in agreement with the fabrication protocol illustrated in Figure 3a.

### 3.4 Analytical performance of the Flower-CN-EC sensor

**FIGURE 4:**
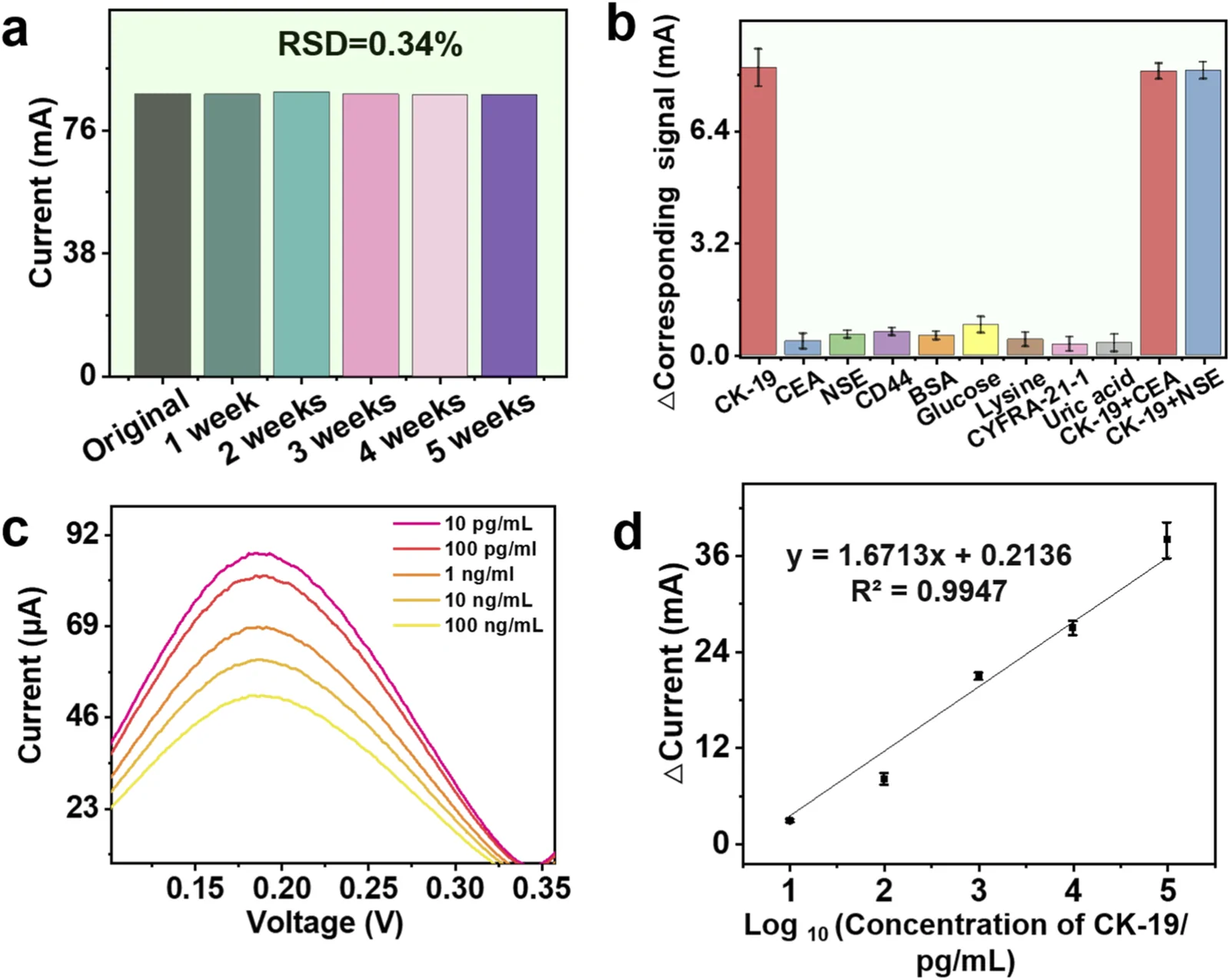
Analytical performance of the Flower-CN-EC sensor for CK-19 detection. **(a)** Storage stability of the Flower-CN-EC sensor over 5 weeks under the tested storage conditions, showing a relative standard deviation, RSD, of 0.34% in the recorded signal response. **(b)** Selectivity evaluation of the Flower-CN-EC sensor toward CK-19 and selected potentially interfering species, including CEA, CA125, CD44, BSA, glucose, uric acid, and CYFRA 21-1. The CK-19 group showed a higher response than the tested interferents under the experimental conditions. **(c)** Differential pulse voltammetry, DPV, responses of the Flower-CN-EC sensor toward different concentrations of recombinant CK-19, showing concentration-dependent changes in peak current. **(d)** Calibration curve derived from the DPV peak-current responses, showing a linear relationship between the electrochemical signal and the logarithm of CK-19 concentration over the tested range from 10 pg/mL to 100 ng/mL. The regression equation was y = 1.6713x + 0.2136, with *R* ^2^ = 0.9947.

The analytical performance of the Flower-CN-EC sensor was evaluated in terms of storage stability, selectivity, linear response, and sensitivity. During five weeks of storage under ambient conditions, the sensor maintained stable responses, with an overall RSD of 0.34% (Figure 4a). This result suggests that the petal-like g-C_3_N_4_-based sensing interface and antibody-modified layer retained good signal stability over the tested period.

Selectivity was assessed by challenging the sensor with CK-19 and several potentially interfering species, including CEA, CA125, CD44, BSA, glucose, uric acid, and CYFRA 21-1. As shown in Figure 4b, the response to CK-19 was clearly higher than those to all tested interferents. Moreover, the presence of uric acid or CYFRA 21-1 caused only minor signal variation, with relative deviations below 5.0% under the tested conditions. Mixed solutions of CK-19 with CEA, uric acid, or CYFRA 21-1 also showed limited interference, indicating that the Flower-CN-EC sensor maintained favorable selectivity in the presence of representative coexisting urinary interferents and related biomarkers. Nevertheless, further interference studies using larger numbers of heterogeneous urine samples will be required to more comprehensively evaluate sensor performance in clinically relevant matrices.

The concentration-dependent response was then investigated using recombinant CK-19 over a range from 10 pg/mL to 100 ng/mL (Figure 4c). As CK-19 concentration increased, the DPV peak current progressively decreased, consistent with the formation of an immunocomplex layer that hindered interfacial electron transfer. By contrast, the signal change, ΔI, increased linearly with the logarithm of CK-19 concentration, yielding the regression equation y = 1.6713x + 0.2136, with *R* ^2^ = 0.9947 (Figure 4d). The limit of detection (LOD) was determined using the 3σ/slope method, where σ is the standard deviation of the blank (n = 10), yielding a value of 3.24 pg/mL (Wang et al., 2022a; Chen et al., 2024; Chen et al., 2025; Li et al., 2025). These results demonstrate that the Flower-CN-EC sensor enables sensitive and quantitative detection of CK-19 over a broad dynamic range.

### 3.5 Prototype visual signal-processing interface

**FIGURE 5:**
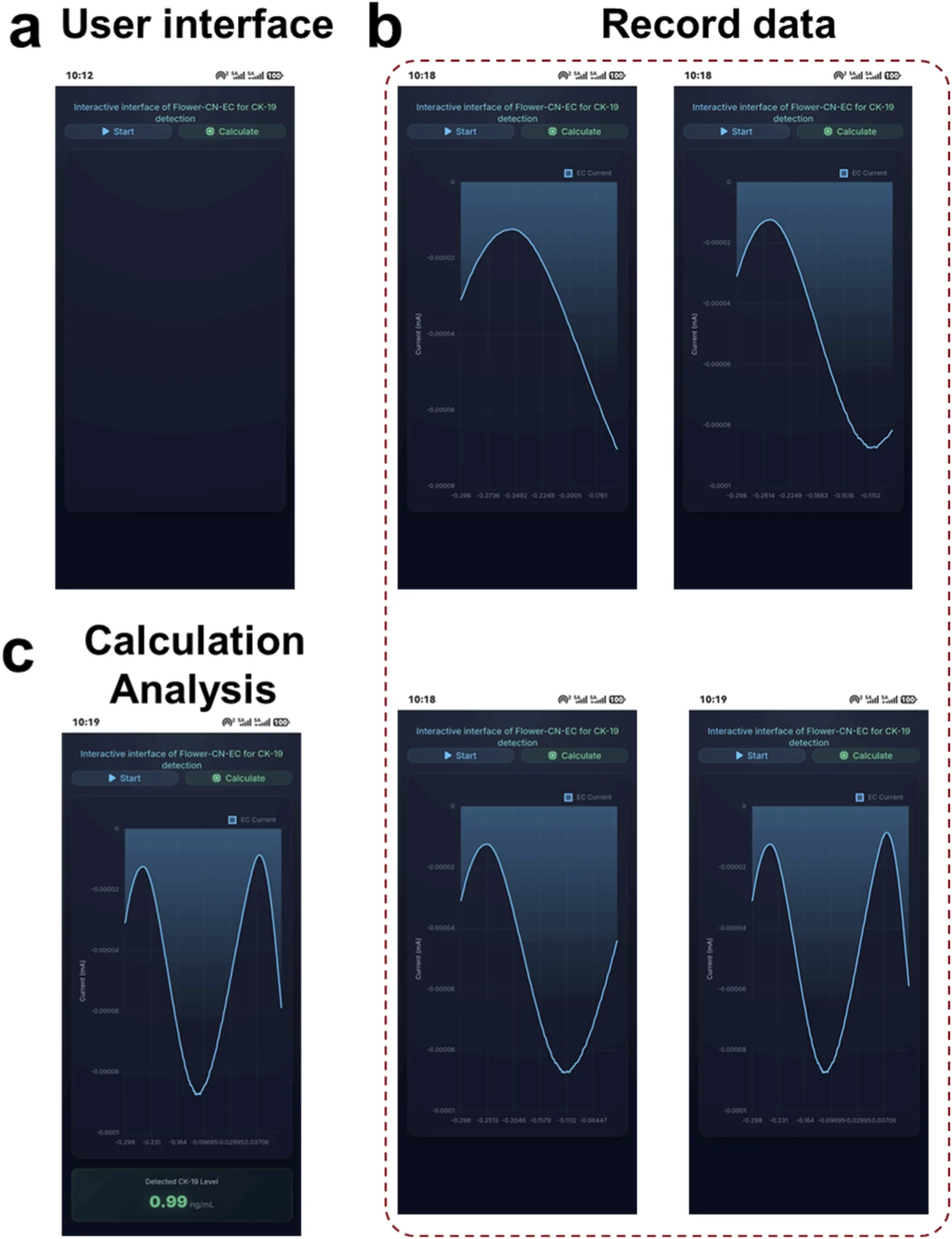
Prototype visual signal-processing interface for Flower-CN-EC-based CK-19 detection. **(a)** Representative screenshot of the user-oriented operation interface, showing the start and calculation functions for CK-19 electrochemical testing. **(b)** Real-time signal-recording pages displaying electrochemical current responses during measurement, allowing visualization and tracking of the raw detection signal. **(c)** Output interface after signal processing, in which the recorded electrochemical response is converted into an estimated CK-19 concentration using a pre-established calibration algorithm. This prototype interface reduces manual data handling and supports future integration of the Flower-CN-EC sensor into portable testing workflows.

To improve usability and facilitate potential translation beyond conventional laboratory settings, an integrated visual detection system was developed for use with the Flower-CN-EC sensor. The interface guides users through the measurement process and presents the detection workflow in an intuitive format (Figure 5a). During testing, raw electrochemical signals are collected and recorded in real time (Figure 5b; Supplementary Video S1). To ensure reliable automated quantification, the system incorporates a dedicated digital signal-processing algorithm. The raw Differential Pulse Voltammetry (DPV) curve undergoes automated baseline correction using a third-order polynomial fitting routine to eliminate charging-current drift. Subsequently, the peak-intensity extraction module automatically isolates the maximum Faradaic current reduction (ΔI). The embedded calibration algorithm then instantly converts this electrochemical parameter into the final urinary CK-19 concentration for user display (Figure 5c). This integrated hardware-software synergy minimizes user-dependent operational errors, enhances data traceability, and provides a robust technological foundation for field-deployable POCT devices. The software prototype was developed in-house using open-source Python libraries (NumPy, SciPy, and Matplotlib) for research and proof-of-concept purposes only, and does not involve any commercial copyright restrictions. It is intended solely for feasibility demonstration under laboratory conditions and has not been validated for standalone clinical diagnostic use.

### 3.6 Pilot evaluation of urine samples from healthy controls and patients with endometriosis

**FIGURE 6:**
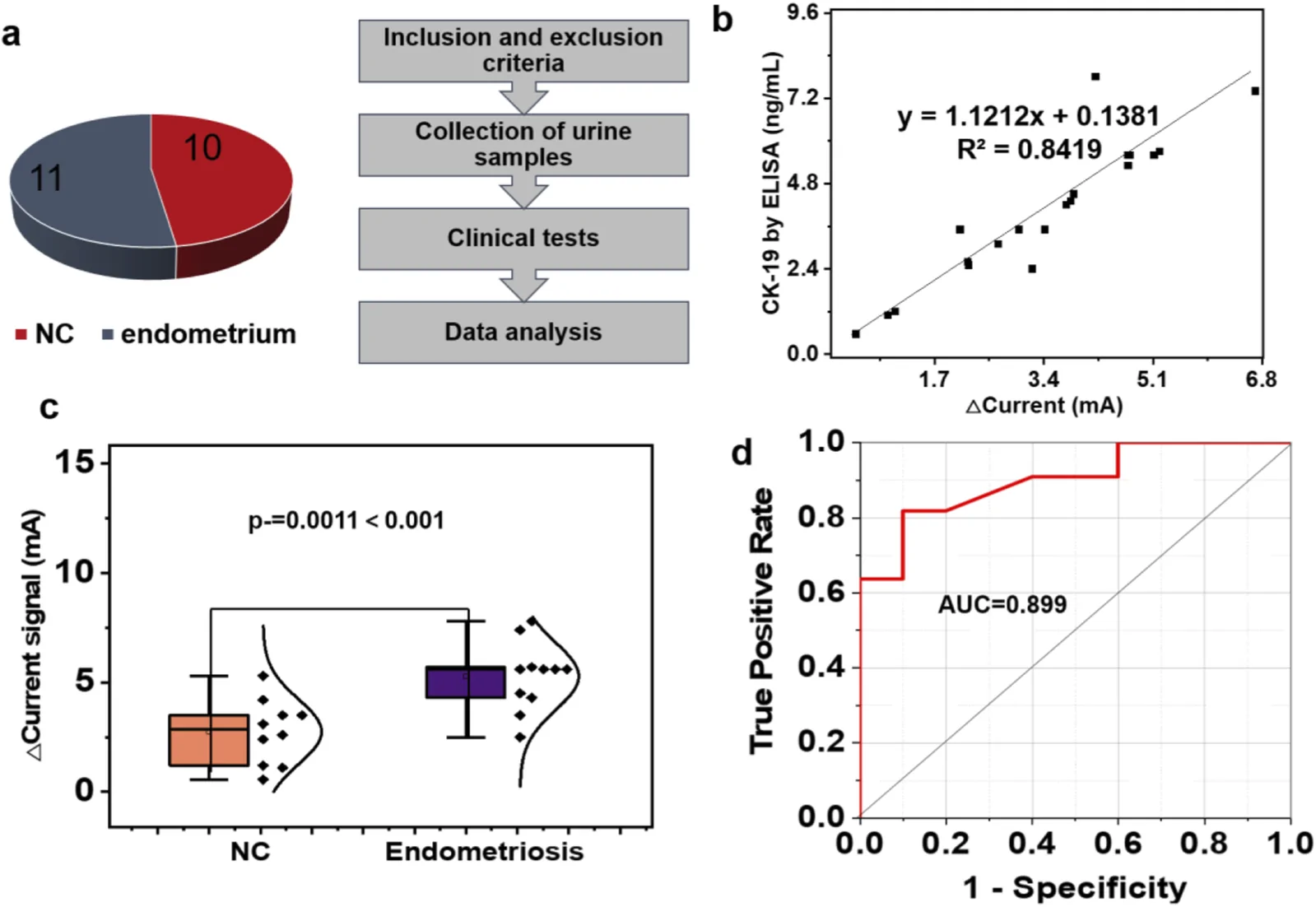
Pilot evaluation of the Flower-CN-EC sensor using human urine samples. **(a)** Composition of the pilot urine-sample cohort, including 10 healthy controls and 11 patients with surgically and histologically confirmed endometriosis. Schematic workflow of the pilot clinical evaluation, including participant screening according to inclusion and exclusion criteria, urine-sample collection, clinical testing, and data analysis. **(b)** Correlation analysis between Flower-CN-EC sensor-derived ΔCurrent signals and ELISA-measured urinary CK-19 concentrations in human urine samples (n = 21). The linear regression equation was y = 1.1212x + 0.1381, with *R* ^2^ = 0.8419, indicating good agreement between the two methods. **(c)** Comparison of sensor responses between the healthy-control and endometriosis groups. The endometriosis group showed higher sensor signals than the healthy-control group in this pilot cohort, with a statistically significant between-group difference, *p* = 0.0011. **(d)** Receiver operating characteristic, ROC, analysis based on sensor signals from the pilot cohort, yielding an area under the curve, AUC, of 0.899. These results suggest preliminary discriminatory potential under the tested conditions, but should be interpreted as exploratory because of the small sample size and the absence of an independent validation cohort.

To preliminarily evaluate the clinical applicability of the Flower-CN-EC platform, urine samples from 10 healthy controls and 11 patients with endometriosis were analyzed (Figure 6a). The detailed demographic and laboratory characteristics of all participants—including age, height, weight, BMI, serum CA125, and AMH levels—are summarized in Supplementary Table S2. The overall testing workflow comprised participant screening, standardized urine collection, sample pretreatment, sensor-based detection, and statistical analysis (Figure 6a). To benchmark the reliability of the Flower-CN-EC sensor, we performed parallel ELISA measurements of urinary CK-19 using the same urine samples. As shown in Figure 6b, the sensor-derived ΔCurrent signals correlated well with ELISA-measured CK-19 concentrations (y = 1.1212x + 0.1381, *R* ^2^ = 0.8419), indicating good agreement between the electrochemical sensor and the standard immunoassay.

The sensor signals obtained from the endometriosis group were significantly higher than those from the healthy-control group (p = 0.0011; Figure 6c). Receiver operating characteristic (ROC) analysis yielded an area under the curve (AUC) of 0.899 (Figure 6d), suggesting promising discriminatory performance in this pilot cohort. However, given the limited sample size and the absence of an independent validation cohort, these findings should be interpreted as preliminary evidence supporting further clinical evaluation rather than as definitive evidence of diagnostic readiness.

## 4 Discussion

### 4.1 Advantages of petal-like g-C_3_N_4_ for electrochemical immunosensing

The petal-like g-C_3_N_4_ architecture played an instrumental role in the construction and superior analytical performance of the proposed electrochemical immunosensor. Compared with bulk g-C_3_N_4_, the petal-like g-C_3_N_4_ architecture provides a 3D hierarchical morphology composed of curled and interconnected nanosheets. This morphology increases the accessible surface area, exposes more interfacial sites for antibody immobilization after plasma activation, and provides open pathways for redox-probe and antigen diffusion. These structural advantages are supported by BET measurements showing an approximately 41% higher specific surface area (Supplementary Figure S2) and by comparative DPV analysis showing more favorable interfacial electron-transfer behavior (Supplementary Figure S3). Collectively, the enlarged accessible surface, improved mass transport, and reduced steric hindrance during antigen-antibody recognition contribute to the high analytical sensitivity of the Flower-CN-EC sensor, including its low LOD of 3.24 pg/mL and good reproducibility with an RSD of 2.31%.

In addition to its morphology, the intrinsic chemical properties of g-C_3_N_4_ are advantageous for biosensor development. The nitrogen-rich framework provides abundant functional sites, while oxygen-containing groups introduced by plasma treatment provide essential anchoring points for the robust covalent conjugation of antibodies via EDC/NHS-assisted coupling, preventing bio-receptor leaching and ensuring long-term interfacial stability. Yadav et al. reported that APTES-functionalized g-C_3_N_4_ electrodes with antibody immobilization via EDC-NHS reaction maintained stability for up to 10 weeks (Yadav et al., 2024). These findings are consistent with our observed five-week ambient storage stability (RSD 0.34%), further validating the robustness of our antibody-modified sensing interface. Moreover, g-C_3_N_4_ is metal-free, chemically stable, low-cost, and compatible with scalable material preparation. The low inter-sensor variation observed in this study further confirms that the petal-like g-C_3_N_4_-modified electrodes can be reproducibly fabricated, establishing a reliable material foundation for future clinical translation and batch production. Compared with previously reported electrochemical sensors for endometriosis biomarkers, which generally employ conventional nanomaterials and rigid electrode formats, the Flower-CN-EC platform uniquely combines a 3D hierarchical g-C_3_N_4_ interface, flexible batch-fabricable electrodes, and a portable visual readout system, thereby offering a more translation-friendly and noninvasive solution that has not been demonstrated in prior electrochemical studies.

### 4.2 Value of noninvasive urinary CK-19 testing in endometriosis monitoring

Noninvasive biomarker testing remains an important unmet need in endometriosis management. Although laparoscopy provides direct visualization and histological confirmation, it is invasive and is not suitable for frequent monitoring. Urine, by contrast, can be collected repeatedly with minimal discomfort and without procedural risk. Therefore, urine-based testing holds immense clinical potential for auxiliary assessment, longitudinal follow-up, treatment-response monitoring, and recurrence surveillance.

In this study, the Flower-CN-EC sensor enabled sensitive CK-19 detection over a broad concentration range and showed highly stable analytical performance. More importantly, urine samples from patients with endometriosis produced significantly higher sensor responses than those from healthy controls. Rather than serving as a stand-alone diagnostic tool at this stage, these findings establish a robust proof-of-concept for utilizing a miniaturized electrochemical platform to capture urinary biomarker fluctuations, paving the way for noninvasive, point-of-care (POC) endometriosis screening.

### 4.3 Positioning of CK-19 as a candidate urinary biomarker

CK-19 is an epithelial cytoskeletal protein, and its aberrant expression or shedding into bodily fluids has been increasingly implicated in the pathogenesis and progression of endometriosis (Tokushige et al., 2011; Carson and Kallen, 2021; Vissers et al., 2024). The present sensor-based findings are consistent with the hypothesis that disease-associated CK-19 fragments undergo renal clearance and can be reliably detected in urine, showing differential levels between individuals with and without endometriosis.

Nevertheless, CK-19 should currently be considered a highly promising candidate urinary biomarker rather than a definitive, stand-alone diagnostic marker. Its routine clinical value requires further validation in relation to disease stage, lesion phenotype, menstrual-cycle phase, hormonal treatment, coexisting gynecological conditions, and inflammatory status. In addition, comparison with established or commonly investigated markers, such as CA125, will be necessary to clarify its incremental diagnostic value. Future studies may also explore whether CK-19 can be integrated into multi-biomarker panels (e.g., combining specific inflammatory cytokines or clinical features) to improve the diagnostic specificity and robustness of noninvasive endometriosis assessment.

### 4.4 Translational considerations and clinical-validation requirements

From a translational perspective, the present work serves as a foundational analytical validation and a proof-of-concept clinical feasibility study. The excellent discriminatory performance observed highlights the platform’s potential utility in distinguishing endometriosis profiles. However, to transition this technology from the bench to the clinic, a substantially higher level of evidence is required.

Future validation should include larger and independent cohorts, preferably from multiple centers, with standardized urine collection, storage, pretreatment, and testing protocols. It will also be important to evaluate sensor performance across different disease stages, lesion subtypes, symptom profiles, and treatment backgrounds. In addition, clinically relevant differential-diagnosis populations should be included, such as patients with adenomyosis, pelvic inflammatory disease, ovarian cysts, uterine fibroids, urinary tract conditions, and chronic pelvic pain without endometriosis. These studies are necessary to determine whether urinary CK-19 sensing can provide clinically meaningful information beyond existing diagnostic workflows.

### 4.5 Limitations and future directions

Several limitations should be acknowledged to guide future research. First, the clinical evaluation was based on a small pilot cohort of 21 urine samples, including 11 patients with endometriosis and 10 healthy controls, which limits statistical power, restricts generalizability, and prevents the establishment of a validated diagnostic cutoff. Therefore, the present clinical results should be interpreted as preliminary proof-of-concept evidence rather than definitive diagnostic validation. Second, potential confounding factors, including age, menstrual-cycle phase, disease stage, lesion subtype, medication exposure, and coexisting gynecological conditions, were not systematically analyzed. Third, although selectivity was confirmed against selected interferents and mixed biomarker solutions, more comprehensive urine-matrix evaluation is needed to assess the effects of variable pH, ionic strength, and high-abundance urinary components. Fourth, while the five-week ambient storage stability supports the preliminary robustness of the sensing interface, further accelerated aging studies and inter-batch validation are warranted. Finally, larger-scale, multicenter studies with independent validation cohorts and clinically relevant differential-diagnosis groups will be essential before the diagnostic utility and clinical applicability of this platform can be established.

In addition, further analytical validation is required using larger numbers of urine samples. Spike-recovery, dilution-linearity, intra-assay precision, inter-assay precision, device-to-device calibration, and operator-to-operator reproducibility should be systematically investigated. Finally, prospective multicenter studies and comparison with established clinical workflows will be essential before the platform can be considered for clinical application. Future work should therefore focus on standardized urine handling, expanded clinical cohorts, independent validation, multi-biomarker integration, and user-oriented testing protocols suitable for POCT scenarios. Additionally, the biological mechanisms underlying CK-19 shedding in endometriosis remain poorly defined. Future studies may incorporate emerging cell niche engineering technologies, such as microfluidic culture systems and micropatterned surfaces, to systematically dissect how biophysical and biochemical cues influence epithelial biomarker release, thereby informing both mechanistic understanding and sensor optimization.

## 5 Conclusion

In summary, we have developed a portable electrochemical immunosensor (Flower-CN-EC) based on a structurally engineered petal-like g-C_3_N_4_ framework for noninvasive urinary CK-19 detection. The 3D hierarchical architecture provides an optimized interface for antibody immobilization and target recognition, enabling ultrasensitive detection (LOD = 3.24 pg/mL) with excellent reproducibility (RSD = 2.31%) and robust ambient stability over 5 weeks. In a pilot clinical cohort, the sensor significantly discriminated endometriosis patients from healthy controls (*p* = 0.0011, AUC = 0.899), establishing the first proof-of-concept for electrochemical urinary CK-19 assessment in endometriosis. This work offers a promising, translation-oriented paradigm for noninvasive endometriosis screening and monitoring, while highlighting the need for larger multicenter validation studies.

## Data Availability

The raw data supporting the conclusions of this article will be made available by the authors, without undue reservation.
