# Supplementary material for: Petal-like g-C3N4-enabled electrochemical immunosensor for urinary cytokeratin 19 assessment in endometriosis: a proof-of-concept pilot study
Source: Front Bioeng Biotechnol. 2026 Aug 27;14:1905091. doi: 10.3389/fbioe.2026.1905091 (PMC13558177; doi:10.3389/fbioe.2026.1905091)
Supplement: Supplementary file 1 [file Supplementaryfile1.docx]

**Petal-like g-C₃N₄-enabled electrochemical immunosensor for urinary cytokeratin 19 assessment in endometriosis: a proof-of-concept pilot study**

Yangyang Wang, MD^a,b,c^; Bin Liu^d,e^; Xiaohua Yu, PhD^a,b,f^; Liu Liu, PhD^a,b,c^; Xiaoying Jin, MD^a,b,c^; Yan Rong, PhD^a,b,c^; Songying Zhang, PhD^a,b,c^*

**Affiliations:**

ᵃ Assisted Reproduction Unit, Department of Obstetrics and Gynecology, Sir Run Run Shaw Hospital, Zhejiang University School of Medicine, Hangzhou 310016, China.

ᵇ Zhejiang Key Laboratory of Precise Protection and Promotion of Fertility, Hangzhou 310016, China.

ᶜ Zhejiang Provincial Clinical Research Center for Reproductive Health and Disease, Hangzhou 310016, China.

^d^ School of Biomedical Engineering, Shanghai Jiao Tong University, Shanghai 200030, China.

^e^ Shanghai 860 Technology Development Company, Shanghai 200438, China.

^f^ Second Affiliated Hospital Zhejiang University School of Medicine, Orthopedics Research Institute of Zhejiang University, Key Laboratory of Motor System Disease Research and Precision Therapy of Zhejiang, Hangzhou 310003, Zhejiang Province, China

*Correspondence: Prof. Songying Zhang, PhD

Assisted Reproduction Unit, Department of Obstetrics and Gynecology, Sir Run Run Shaw Hospital, Zhejiang University School of Medicine, No. 3 East Qingchun Road, Hangzhou 310016, China.

Tel: +86-571-86002220

E-mail: zhangsongying@zju.edu.cn

**S1.** TEM analysis of C_3_N_4_.

**Figure S2.** Nitrogen adsorption-desorption isotherms of petal-like g-C₃N₄ and bulk g-C₃N₄. The petal-like g-C₃N₄ showed a higher BET specific surface area than bulk g-C₃N₄, supporting the formation of a more accessible 3D nanosheet-based interface.

**Figure S3.** Comparative differential pulse voltammetry (DPV) responses of petal-like g-C₃N₄- and bulk g-C₃N₄-modified electrodes in [Fe(CN)₆]³⁻/⁴⁻ solution, indicating more favorable interfacial electron-transfer behavior at the petal-like g-C₃N₄ interface.

**Table S1. Spike recovery analysis of Flower-CN-EC.**

| **Spike solution number** | **CK-19** | **Spiked recovery (%)** |
| --- | --- | --- |
| 1 | 100 | 102.1 |
| 2 | 100 | 101.2 |
| 3 | 100 | 101.3 |
| 4 | 100 | 98.2 |
| 5 | 100 | 98.2 |
| Average (n=5) |  | 100.200 |
| SD |  | 1.859 |
| RSD |  | 3.487 |

**Table S2. Detailed information for clinical biosamples.**

| **Number** | **Group** | **Age** | **Height (cm)** | **Stage** | **Menstrual phase** | **Weight (kg)** | **BMI** | **CA125 (U/mL)** | **AMH (ng/mL)** |
| --- | --- | --- | --- | --- | --- | --- | --- | --- | --- |
| 1 | Endometriosis | 34 | 155 | 2 | No | 60 | 24.974 | 10.59 | 0.74 |
| 2 | Endometriosis | 38 | 162 | 1 | No | 52 | 19.814 | 11.12 | 1.99 |
| 3 | Endometriosis | 30 | 161 | 3 | No | 61 | 23.533 | 89.22 | 3.26 |
| 4 | Endometriosis | 28 | 163 | 4 | No | 53 | 19.948 | 94 | 2.37 |
| 5 | Endometriosis | 35 | 160 | 2 | No | 45 | 17.578 | 24.73 | 4.44 |
| 6 | Endometriosis | 33 | 163 | 4 | No | 45 | 16.937 | 81 | 0.65 |
| 7 | Endometriosis | 36 | 156 | 3 | No | 52.5 | 21.573 | 95.59 | 0.75 |
| 8 | Endometriosis | 37 | 167 | 4 | No | 51 | 18.287 | 54.28 | 1.2 |
| 9 | Endometriosis | 30 | 163 | 2 | No | 45 | 16.937 | 30.5 | 1.42 |
| 10 | Endometriosis | 39 | 161 | 1 | No | 55 | 21.218 | 23 | 0.3 |
| 11 | Endometriosis | 33 | 162 | 3 | No | 56 | 21.338 | 23.8 | 3.62 |
| 12 | Control | 31 | 160 | / | No | 45 | 17.578 | 7.24 | 0.66 |
| 13 | Control | 37 | 150 | / | No | 54 | 24.000 | 13.84 | 4.46 |
| 14 | Control | 37 | 157 | / | No | 70 | 28.399 | 11.11 | 3.89 |
| 15 | Control | 37 | 162 | / | No | 67.5 | 25.720 | 17.15 | 2.05 |
| 16 | Control | 35 | 150 | / | No | 70 | 31.111 | 16.81 | 3.31 |
| 17 | Control | 29 | 160 | / | No | 61 | 23.828 | 9.71 | 9.92 |
| 18 | Control | 31 | 153 | / | No | 49 | 20.932 | 10.25 | 9.6 |
| 19 | Control | 31 | 165 | / | No | 53 | 19.467 | 14.26 | 2.9 |
| 20 | Control | 31 | 164 | / | No | 60 | 22.308 | 14.5 | 2.41 |
| 21 | Control | 28 | 160 | / | No | 53 | 20.703 | 27.97 | 3.86 |
